# Addressing disparities in pharmacogenomics through rural and underserved workforce education

**DOI:** 10.3389/fgene.2022.1082985

**Published:** 2023-01-16

**Authors:** Jacob T. Brown, Erin McGonagle, Randall Seifert, Marilyn Speedie, Pamala A. Jacobson

**Affiliations:** ^1^ Pharmacy Practice and Pharmaceutical Sciences, University of Minnesota College of Pharmacy, Duluth, MN, United States; ^2^ Experimental and Clinical Pharmacology, University of Minnesota College of Pharmacy, Minneapolis, MN, United States; ^3^ Pharmaceutical Care and Health Systems, University of Minnesota College of Pharmacy, Minneapolis, MN, United States; ^4^ Medicinal Chemistry, University of Minnesota College of Pharmacy, Minneapolis, MN, United States

**Keywords:** pharmacogenomics, pharmacogenetics, rural, underserved, education

## Abstract

**Introduction:** While pharmacogenomic (PGx) testing is routine in urban healthcare institutions or academic health centers with access to existing expertise, uptake in medically-underserved areas is lagging. The primary objective of this workforce education program is to extend access to didactic, case-based and clinical PGx training for pharmacists serving rural Minnesota and populations experiencing health disparities in Minnesota.

**Methods:** A PGx workforce training program funded through the Minnesota Department of Health was offered through the University of Minnesota College of Pharmacy (COP) to pharmacists working in rural and/or underserved areas in the state of Minnesota. Learning activities included a 16-week, asynchronous PGx didactic course covering PGx topics, a 15-min recorded presentation, an in-person PGx case-based workshop, and a live international PGx Conference hosted by the University of Minnesota COP and attendance at our PGx Extension of Community Health Outcomes (ECHO).

**Results:** Twenty-nine pharmacists applied for the initial year of the program, with 12 (41%) being accepted. Four (33%) practiced in a hospital setting, four (33%) in retail pharmacy, two (17%) in managed care, and two (17%) in other areas. The majority had not implemented a PGx program as part of their practice, although nearly all responded definitely or probably yes when asked if they expected their organization to increase its use of PGx testing services over the next three years. All participants either strongly or somewhat agreed that this program helped them identify how and where to access clinical PGx guidelines and literature and improved their ability to read and interpret PGx test results. Eight participants (67%) strongly or somewhat agreed that they expected to increase the number of PGx consultations in their practice, while ten (83%) strongly or somewhat agreed they would be able to apply what they learned in this program to their practice in the next six months to a year.

**Discussion:** This novel PGx training program focused exclusively on pharmacists in rural and/or underserved areas with a delivery method that could be accomplished conveniently and remotely. Although most participants’ organizations had yet to implement PGx testing routinely, most anticipated this to change in the next few years.

## Introduction

Precision medicine is an innovative approach to preventing and treating diseases using an individual’s genetic makeup and lifestyle (Jameson and Longo, 2015). Pharmacogenomics (PGx) is one of the most developed forms of precision medicine, one that has moved beyond discovery and into clinical care (Roden et al., 2019). PGx provides additional information that can help predict the most effective medication or those with the lowest risk of side effects. A large body of scientific evidence has shown that genetic variation within genes involved in the metabolism or the mechanistic pathways of medications is important and is sufficiently robust for implementation into practice (Haidar et al., 2022).

There are several barriers that limit widespread implementation of PGx, despite its potential in improving medication therapy management and reducing costs (Haidar et al., 2022). PGx is not yet used in most rural healthcare organizations and in those primarily caring for people from underrepresented groups, in part due to the complexity of genetic interpretation and a lack of a healthcare workforce trained in its use. While other factors surely contribute to this, such as lack of resources and prioritization of other unmet healthcare needs, the absence of individuals trained in PGx contributes to the lack of PGx-guided medical care. Focused and intentional education in PGx is needed to reduce and eliminate the growing health disparities occurring around application of PGx.

Effective use of PGx information in the care of patients requires pharmacists to be adequately versed and confident in the use of genomics, understand the relationship between genomics and drug action, understand when and how PGx should be applied, and be able to communicate the benefits and limitations of PGx to their patients and other health professionals (Kennedy, 2018). As a profession, pharmacy is preparing its workforce for PGx-guided healthcare and education of the workforce in rural areas and those practicing in underserved populations is particularly critical for several reasons. Pharmacists who completed their training prior to 2010 would have had little, if any, formal PGx education and rural pharmacists are more likely to be older relative to those in urban areas (Minnesota Department of Health, 2019a). Pharmacists in underserved areas have historically practiced at the top of their license and are more likely to have decision making authority that provides opportunities for them to order and apply PGx information. Patients in underserved areas are also more likely to be from diverse groups, which may have a higher frequency of certain at-risk genetic variants (e.g., *CYP2D6*, *HLA*) and make the utility of PGx of even greater value.

The University of Minnesota College of Pharmacy, with funding from the Minnesota Department of Health, has developed a certificate granting PGx training program aimed at practicing pharmacists in rural and underserved populations in the state of Minnesota. Several unique underserved communities reside in Minnesota, such as American Indian, US Hmong, Karen and Somali. In addition, nearly 10% of its population live in rural areas (Bishop et al., 2021). The primary objective of this program is to expand PGx pharmacist education to those practicing in rural areas and to those working with underserved populations.

## Materials and methods

**FIGURE 1:**
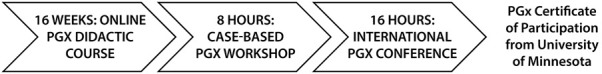
PGx training program timeline.

A PGx training program was offered through the University of Minnesota College of Pharmacy to Minnesota licensed pharmacists working in rural and/or underserved areas in the state of Minnesota (Figure 1). Applicants were considered to be serving a rural population if they were outside of the Minneapolis-St. Paul metropolitan and Duluth areas, or if the primary population they served extended to rural areas. Underserved populations were defined as those groups experiencing health disparities which could be in urban or rural areas of Minnesota. This certificate-granting program was aimed towards pharmacists interested in improving health equity in Minnesota by advancing clinical PGx, those who have not been in pharmacy school recently or did not cover PGx in their pharmacy learning, those who face challenges in accessing existing PGx expertise and training opportunities, and those who can devote sufficient time and effort for approximately nine months to learn about PGx principles and PGx guided healthcare. The certificate was focused on practicing pharmacists given their role as medication experts and their ability to incorporate PGx into their clinical practices. Applicants were recruited through state pharmacy organizations, College of Pharmacy alumni channels, and the Minnesota Board of Pharmacy. A formal review committee was established of pharmacy faculty to review and score applications. Individual applications were reviewed based on 1) working with rural and/or underserved populations, 2) having a practice area relevant to PGx with future potential to apply PGx, 3) personal interest and motivation to learn PGx, 4) quality of their written personal statement and, 5) commitment to complete the full program. The 12 applicants with the highest scores were offered admission.

The overall objectives of the PGx workforce training program are shown in Table 1. The program included a 16-week, asynchronous PGx didactic course (Applied PGx for the Healthcare Workforce) covering several PGx topics with homework assignments; creation of individual 15-min recorded presentations on a PGx topic selected by the learner; and passing a comprehensive open-resource final assessment. Learners also participated in a full-day, in-person, case-based PGx training and a virtual two-day international PGx Conference hosted by the University of Minnesota College of Pharmacy. They were also required to attend at least one PGx Extension of Community Health Outcomes (ECHO) session hosted by the College of Pharmacy. ECHO sessions are live, online presentations by clinicians of real-world cases for which they are seeking input on whether PGx testing would be beneficial, how to interpret the results, and how they can be utilized for patients. Participants successfully completing the training received a PGx certificate of participation from the University of Minnesota, a stipend for their time, travel/lodging costs for one in-person event, registration and continuing education (CE) from the conference.

**TABLE 1:** Learning objectives of the University of Minnesota PGx workforce educational program.

Upon completion of program, trainees are able to
• Understand basic genetics tailored to the clinician
• Identify how and where to access clinical PGx guidelines and literature
• Know how to read and interpret PGx test results, and know when and how to apply results in patient care;
• Understand how differences in genetic variability across ancestry groups applies to PGx
• Assess PGx testing assays and be able to select the test panel relevant for a patient population
• Understand the limitations of PGx testing
• Understand how drug-drug interactions may be altered in severity by genetic variability and know how to modify therapy;
• Describe cost of PGx testing and current reimbursement issues
• Demonstrate how to document PGx results and implications, and how to explain PGx results and implications of the information to patients
• Understand the ethical, legal, and social issues in handling of PGx data; and
• Demonstrate how to orally communicate PGx to other healthcare providers

The clinical didactic course presented the concepts of the science underpinning PGx, exploring how pharmacogenes are known to affect human therapeutics and highlighted content related to clinical practice. These topics included an introduction to the human genome and pharmacogenes, clinical PGx resources and guideline (i.e., Clinical Pharmacogenetics Implementation Consortium (CPIC)) development, and PGx in psychiatry, opioids, proton pump inhibitors, NSAIDs, cardiology, oncology, HLA, and pediatrics. Additional topics included how PGx pertains to genetic exceptionalism; health equity; PGx testing panels; educating patients in PGx; regulatory considerations; ethical, legal, and social issues; clinical decision support and implementation of PGx in a large health system (Table 2). Participants were required to deliver and record an oral presentation on a PGx topic in their interest area with faculty and fellow learner feedback in order to develop their ability to communicate effectively about PGx.

**TABLE 2:** Topics covered in online didactic course, clinical case workshop and national conference sessions.

Online didactic course	Clinical case workshop	Conference sessions
• Introduction to the human genome and pharmacogenes	PGx cases in	• Economic value of PGx in clinical care
• Clinical PGx resources and CPIC guidelines	• Mental health	• Overcoming economic barriers in PGx
• How PGx pertains to genetic exceptionalism	• Cardiology	• Use of PGx in preventing harm
• PGx and health equity	• Pediatrics	• Costs of implementing health system-wide PGx
• PGx testing panels	• Oncology	• Buying *versus* building clinical decision support tools for PGx
• Educating patients in PGx	• Bone marrow transplant	• Payer perspectives on PGx
• Regulatory considerations of PGx	• NSAIDs	• Local coverage determinations for PGx testing and reimbursement
• ELSI and PGx	• Opioids	• Integrating medication therapy management/PGx in practice
• PGx clinical decision support	• Proton pump inhibitors	• Educating patients in PGx and return of results
• Implementation of PGx in health systems	• PGx assays and phenotype calls	• Challenges and solutions for PGx in rural and underserved populations
• PGx in cardiology	• CYP2D6 phenoconversion	• Updates to PGx guidelines for SNRIs and SSRIs, beta blockers, statins, NSAIDs and clopidogrel
• PGx in oncology		• Complex PGx associations/multiple drug-metabolizing genes and pharmacodynamic genes
• PGx in pediatrics		• Regulatory issues relevant to PGx
• PGx in psychiatry		• Developing telehealth for PGx
• PGx in HLA		• Novel education and implementation strategies for PGx
• PGx in NSAIDs		• Legal/liability implications of PGx for physicians and pharmacists
• PGx in opioids		• Moving beyond Eurocentric PGx to improve health outcomes for all
• PGx in proton pump inhibitors		

The national PGx conference was a CE-bearing international conference which has been hosted by the College of Pharmacy every other year since 2016. The first part of the conference was a one-day in-person clinical case workshop (6.75 h), while learners also attended a 2-day conference following the case workshops which could be attended in-person or virtually (12.25 h). Topics and drugs covered in the workshop and conference are shown in Table 2. Presenters were clinical experts and/or practitioners in the field using PGx, those with services important to support PGx, and/or those who have successfully implemented PGx into their practices.

At the conclusion of the didactic portion of the program, participants were asked to complete a short survey developed by the program staff and faculty to assess the program, changes in their own PGx knowledge and attitudes, expected changes to their clinical practice based on their participation, and current barriers to PGx implementation in their current practice setting.

## Results

**FIGURE 2:**
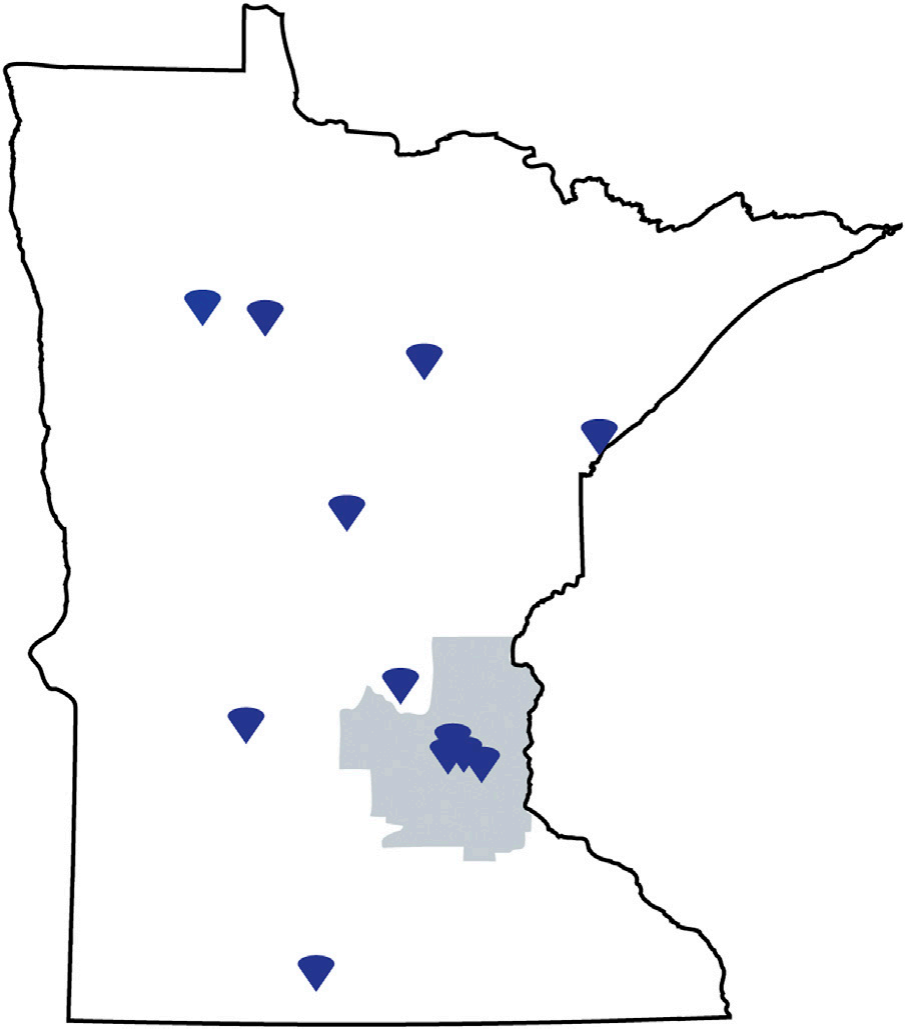
State of Minnesota map indicating location of learners practice site.

A total of 12 out of 29 applicants were accepted into the inaugural program and all completed the certificate program. The next cohort begins in January 2023. Common reasons applicants scored low by the admission committee were not working with a rural or underserved population, not practicing in an environment that would allow for implementation of PGx, or having a personal statement that did not strongly describe why they wanted to participate in the program. All participants had a PharmD degree. Four (33%) reported working in a hospital pharmacy, four (33%) in community pharmacy, one (8%) in a clinic, two (17%) in managed care, and two (17%) as “other.” As seen in Figure 2, participant’s practice areas were located throughout the state of Minnesota. Those outside the Minneapolis-St Paul metro area were rural pharmacists and those within the metro area were caring for underserved populations.

Prior to starting the program, when asked if their clinical practice had implemented a PGx program, eight (67%) responded No, two (17%) responded Yes, a small pharmacy-led program, and three (25%) noted they were currently in the process of implementing a PGx program. When asked if their organization used PGx in clinical care, four (33%) responded Yes, seven (58%) No, and one (8%) that they did not know. Half of participants stated their organization ordered fewer than 10 PGx tests per year, one that they ordered 100 or more, and five stated they did not know. Similarly, when asked how often they use PGx-guided care in their practice, half responded Infrequently, four stated Never, one Monthly, and one Weekly.

**FIGURE 3:**
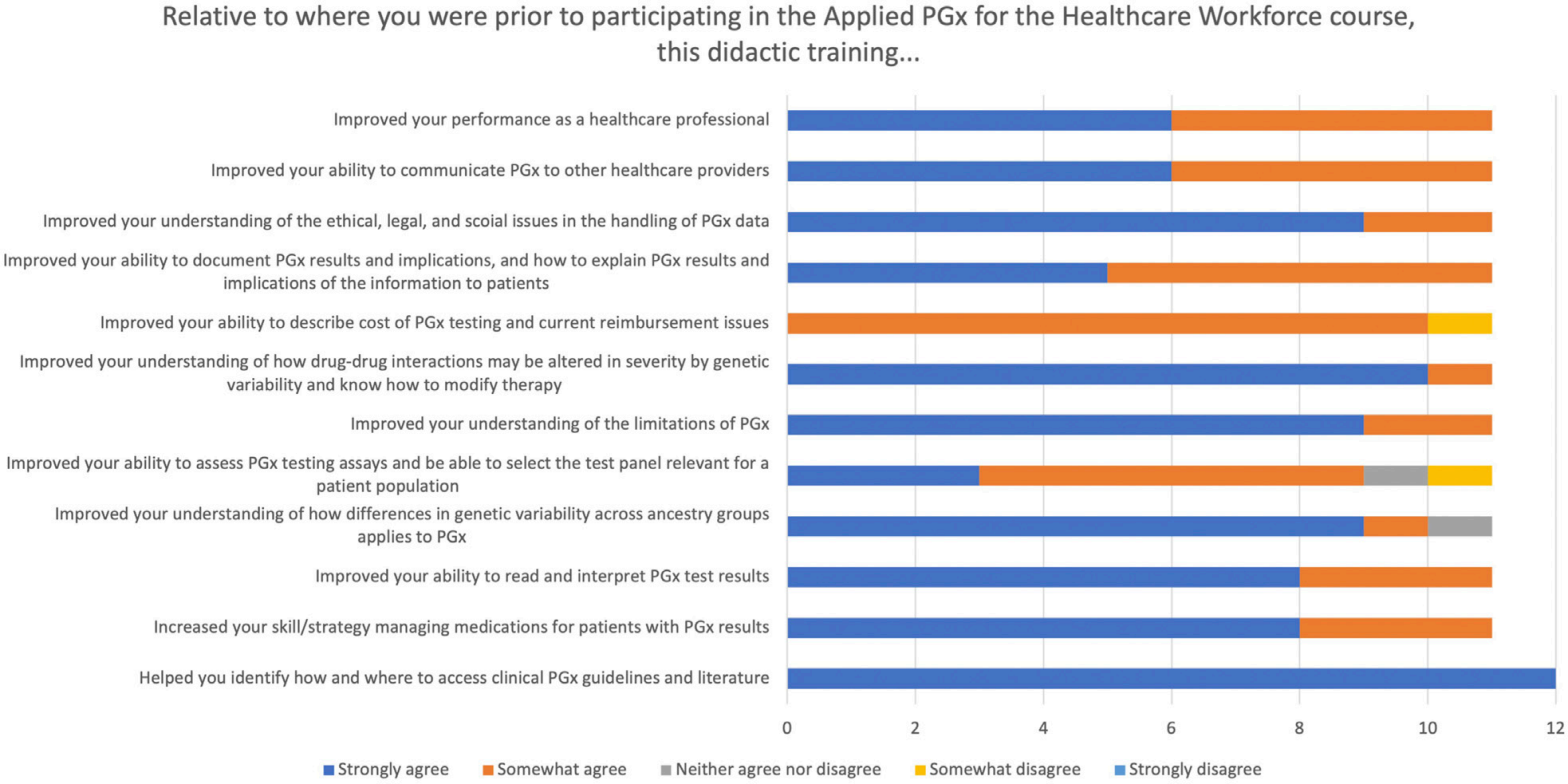
Participant responses to the didactic course evaluation.

**FIGURE 4:**
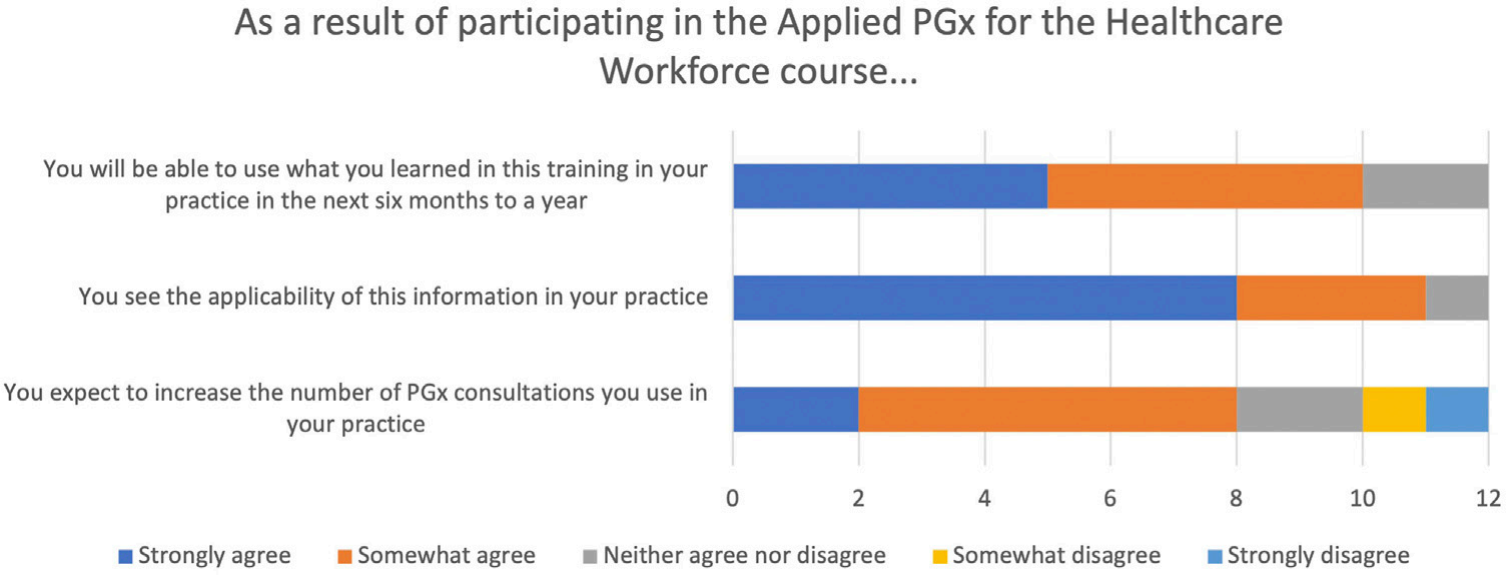
Participant responses relative to PGx practice after completing the didactic course.

Responses related to an individual’s level of understanding and future plans for PGx are shown in Figures 3, 4. Compared to where learners were prior to participating in the course, the majority strongly or somewhat agreed their understanding of multiple PGx issues was improved as well as their ability to communicate PGx to other healthcare providers and patients. Nearly all learners strongly or somewhat agreed they saw the applicability of PGx in their practice and they would be able to use the information learned in the next 6 months to a year.

Participants were also invited to provide written feedback, which included the following from separate individuals.

• “*I recommend (it) to any pharmacist wishing to learn more about this exciting aspect in pharmacy.”*
• *“It was a great experience and I learned a lot.”*
• *“Gaining knowledge of the available resources will allow me to continue to implement changing guidelines in my current practice. The PGx ECHO sessions (attended several due to interest!) were really great - being able to discuss real patient cases with clinicians currently practicing with PGx data was invaluable.”*
• *“Overall this was a great course. I was extremely grateful to have the opportunity to learn more about PGx in this setting. I feel it really helped to catch me up on information that was not taught during my time in a PharmD program. It was something I was interested in, but would have found overwhelming to self-teach without this base.”*
• *“Loved the opportunity to participate in this course. Most of it was real word applicable, held my interest, was applicable to patient care, and didn’t feel like busy work or straight memorization.”*

Participants were asked about what barriers in their clinical practice could hinder their ability to implement what they learned. These included resources devoted to the scalability of implementing PGx, insurance coverage/reimbursement of testing and appointments with a pharmacist, lack of access to patient medical records/PGx test results, lack of organizational interest, and current level of evidence for prior authorization criteria and exceptions.

One unique challenge for this first cohort was the timing in regards to the global COVID-19 pandemic. Pharmacists in rural areas in particular struggled at times to balance staffing shortages during a surge in cases with the demands of completing the program. Faculty teaching in the course also teach PGx content in the PharmD program and a programmatic challenge was adapting lectures intended for delivery to students for working professionals.

## Discussion

PGx is a promising innovation to improve medication safety and efficacy, but it will not be fully realized until the broader healthcare workforce is adequately prepared. It is vital that PGx not be available only to those in larger metropolitan areas from large healthcare organizations, but to intentionally and strategically create programs for pharmacists caring for rural and underserved populations. This PGx workforce training program of pharmacists is unique in that it includes not only lectures from PGx content experts, but also includes a full day of case-based learning, participation in a novel PGx ECHO program, and a two-day PGx conference with presentations from PGx experts. As shown by survey of the first cohort to participate in this certificate, their overall education level increased, with future plans to offer or enhance PGx guided care at their respective institutions.

Knowledge and attitudes of PGx in healthcare professionals is well described, with most individuals acknowledging the importance and value of PGx but also expressing their own discomfort in interpreting and applying results (Kennedy, 2018; Olander et al., 2018; Frigon et al., 2019; Hundertmark et al., 2020). The American Association of Colleges of Pharmacy PGx Special Interest Group recently updated core competencies in genomics for pharmacists, which are reflective of the learning objectives of this program (Gammal et al., 2021). Our program is novel as compared to other PGx education programs in several ways: 1) the primary focus was on equitable access to education: learners were offered stipends for their time rather than charging a fee so that they could take unpaid time off, get childcare, *etc.,*, in order to participate. Employers were also not expected to pay for their education to minimize the imbalance for well-funded vs. underfunded organizations, 2) the program is attempting to create over time a community of PGx-trained practitioners throughout the state by using a cohort model and by requiring PGx ECHO and conference participation, 3) it is offered by a public, land-grant university rather than a professional organization.

To date, the majority of large PGx implementation programs have been limited to larger institutions (Luczak et al., 2021a). PGx in rural or non-metropolitan areas has received little attention overall, where the opportunities, challenges, and barriers are different (Dressler et al., 2019). Approximately a decade ago Dorfman et al. conducted interviews in western Montana with 17 clinicians, including physicians, a pharmacist, nurse practitioners, registered nurses, and a physician’s assistant, practicing in rural areas and tribal clinics assessing their views on PGx testing. Participants had a wide range of practice experience as well as comfort level with PGx testing. Providers were generally optimistic about the potential for PGx to improve outcomes, although had concerns about other priorities (e.g., medication adherence) before PGx testing, turnaround time, and testing accessibility. Notably, since the time of these interviews over 20 CPIC guidelines have been published and/or updated, and the number of companies offering PGx testing has increased. Other barriers remain true today, such as cost, insurance reimbursement, and uniquely to these interviews concerns from American Indian/Alaska Native communities acceptance around genetic testing (Dorfman et al., 2015).

While Dorfman et al. focused on providers working in rural areas, Stegelmeier et al. 7-item survey focused on adult individuals filling a prescription at a community pharmacy in a rural area. A total of 52 individuals completed the survey. The clear majority in this group were taking a prescription medication, while average responses reflected a neutral to agree response in regards to their understanding of what PGx is, their interest in PGx, and a belief that PGx could help them. On average, this group expressed they would be willing to pay just $51 out of pocket for PGx testing, but generally did agree that PGx testing is valuable (Stegelmeier et al., 2020). While it may be difficult to broadly generalize these results to all rural populations, it does provide an informative starting point for programs looking to implement/offer PGx testing in more rural areas. As shown in Figure 2, our education program was able to successfully target and enroll individuals from rural areas, with approximately half of participants in the first cohort either located in or serving a rural population.

Although Minnesota has excellent healthcare, it also has considerable health disparities between whites and people of color and American Indians (Minnesota Department of Health, 2019b; Bishop et al., 2021). Reaching and educating the pharmacists and other healthcare clinicians working with these underserved populations is critical to avoid worsening of existing health disparities. Khoury et al. (2022) recently described the need for a public health approach to health equity in genomics and precision medicine, and much of the same can be said of PGx.

Beyond clinical implementation, research efforts to identify novel variants that may impact PGx are also needed Luczak et al. (2021b). describe approaching PGx research and translation with an equity lens to populations historically excluded from this type of research. It is important to note that the majority of the research informing our current knowledge of PGx comes from populations with European ancestry, and there may be gaps in knowledge when applying this to individuals of non-European ancestry. Fohner et al. (2019) acknowledge several challenges in working with rural and underserved populations and provide several recommendations to overcoming these challenges. More recently, Leitch et al. conducted interviews related to the utility of PGx with various healthcare stakeholders from three different institutions in Montana that serve neglected populations (Leitch et al., 2022). While they expressed similar sentiments of positive perceived value of PGx and barriers to testing, they did note the potential of providing PGx integration through telehealth visits. Our educational program specifically addressed the limitations and cautions of PGx knowledge when applying it to underserved population who are more likely to be of non-European ancestry.

In February of 2022, the Right Drug Dose Now Act was introduced to the United States Congress, with the purpose of updating the National Action Plan for Adverse Drug Event Prevention (Right Drug Dose Now Act, 2022). This bill proposes to provide additional education on PGx to the public as well as healthcare professionals, and specifically mentions rural and medically underserved communities. A program such as ours could serve as a model for education delivered with funding from this bill. The next step for our program, which is currently supported by the Minnesota Department of Health, is to train an additional 23 pharmacists practicing in rural areas or underserved populations within Minnesota. While originally we intended to enroll three cohorts over three years, the interest and demand for the program dictated that we combine the second and third cohorts into one to accelerate training.

Adequate PGx education of clinicians, including pharmacists, physicians, nurse practitioners, physician assistants, and registered nurses will be critical moving forward as PGx continues to be implemented. In order to avoid worsening rural/urban health disparities, and those seen in underserved populations, it is critical that this education extend to all healthcare professionals working with these populations. Directly engaging these communities in PGx certificate training programs is one method to increase the knowledge and understanding of not only foundational PGx knowledge but also PGx implementation so all individuals can benefit from PGx informed care.

## Data Availability

The raw data supporting the conclusion of this article will be made available by the authors, without undue reservation.
